# On a New Mode of Applying Atropine

**Published:** 1864-05

**Authors:** Julius Homberger


### ON A NEW MODE OF APPLYING ATROPINE.

By JULIUS HOMBERGER, M.D.

In the last number of the first volume of this Journal I have alluded to the efficacy of applications of sulphate of atropine, in substance, into the eye, in all those cases which indicate otherwise repeated instillations. The use of solutions of atropine, as well as atropine ointment, and even Streatfield's atropine paper, will, I think, henceforth be limited to the dilatation of the pupil in ophthalmoscopic examinations. I have since that time experimented with atropine on all my patients in the solid form only, and I must state that the results have been such as to justify the most enthusiastic recommendations. The dilatation of the pupil produced in eyes without disease of the iris, in cases of keratitis, wounds of the cornea, etc., etc., lasts usually from four to five days. The quantity of atropine placed into the lower palpebral sac seems to have a decided influence on the duration of dilatation of the pupil. The following is an instance of this long action: By mistake, I had put atropine in the sound eye of a patient, who applied for treatment, and when, fifteen minutes later, I found the error, the pupil was considerably dilated. I applied the atropine to the diseased eye, and, in order to neutralize the effects of atropine on the good one, I put a square of Calabar bean paper in the latter. The pupil became smaller, after about twenty minutes; but the patient returned in the evening, with a pupil as large as before. I sacrificed, during the succeeding three days, eight squares of the Calabar bean paper, which is, at the present moment, such a treasure in this country as not to be freely dealt with, and the dilatation always returned, and had only entirely disappeared when I saw the patient on the seventh day. There is no doubt that the large quantity of atropine, taken endosmotically into the humors of the eye, was the cause of this remarkably long-lasting effect; and I suppose that the rapid dilatation of the pupil, by detachment of posterior synechies, which I obtained both in cases of chronic and acute iritis, with not more than one application daily, must be attributed more to the reception of the alkaloid into humors and tissues, from where it exerted a continual action on the iris, than to the sudden effect of a high dose of the remedy. That the active principles of the Calabar bean contract the pupil momentarily, may be easily explained. It may be supposed that the endosmotic process is more active, through the cornea than through the sclerotic, and that, therefore, the atropine contained in the aqueous humor is neutralized, and, after having received an over-balancing quantity of the active principles of Calabar bean, acts on the iris; while, after some time, the larger quantity of atropine, yet contained in the vitreous humor and the other tissues, neutralizes the Calabar bean, and again produces dilatation of the pupil. I have seen that, within two days, and with only one application of atropine daily, large adhesions of the iris were torn from the anterior capsula, sometimes leaving there intensely black spots, and effused lymph, the former of which derived their origin from the uveal surface of the iris. In one case of syphilitic iritis, two *nodi*, situated near the pupillar margin, and which had resisted the first applications of atropine, though the rest of the iris had been considerably retracted, were removed, about one line towards the periphery, on the fifth day. This is the more remarkable, as their size had not diminished during that period, and the inflammation was yet considerable.

It cannot be questioned that it is much easier to introduce a solid particle into the eye, than to introduce a square bit of paper; that the latter can sometimes not be removed without the aid of forceps; that the lids of iritic patients have, sometimes, to be kept open for a considerable time, while the paper is removed, in spite of the patient's sensitiveness to light; and that in a certain number of cases the patients complain of the slight irritation produced by the paper. While these little inconveniences do not appertain to the application of atropine in substance, which is rapidly dissolved, and felt only for a moment as a foreign body, I think, particularly, the circumstance that it is possible to conveniently saturate the tissues and humors of the eye, with the remedy, will render this method also of greater therapeutic value.

It may be set down as a fundamental point in the treatment of iritis that the dangers of this disease, for vision, consist *merely in the formation of adhesions between iris and anterior capsula.* During the acute stage of the disease, the adhesions threaten to glue the pupillary margin to the capsula. Either, if lymph is effused into the pupillary space, the eye becomes useless by closure of the pupil, after the acute symptoms have subsided, or the synechies become the points of origin of a chronic process, which very frequently leads to a number of complicating changes in the choroid, ciliary body, and lens, almost certainly fatal to vision.

The danger of the formation of synechies is, therefore, of primary importance in the treatment of acute iritis; in fact, a case of this disease can only be considered as cured if no adhesions are left after the inflammation is gone. The first object of the oculist treating this malady must therefore be to dilate the pupil, at all hazards. To rely on the removal of adhesions by constitutional medication, and to be satisfied with a mercurial treatment, with instillations of atropine three or four or six times daily, would not be judicious, in my opinion. The application of the mydriatic *coup sur coup* is the source of considerable irritation to the eye; and, on the other hand, the resorption of the fluid, with the tears, through the lachrymal canal, exposes the patient to the danger of poisoning, to the same extent, as the application of atropine in substance.

The method of treating iritis, which I would propose, consists in the introduction of the fortieth part of a grain of atropine, in substance, into the lower conjunctival sac, which can be easily done by placing the salt, with a probe, on the everted lower lid. The patient is kept for half an hour under observation. Dryness in the throat is a usual effect of the application of the drug, which soon passes away; only if further symptoms (congestion to the head, paralysis of the m. protrusor urinæ) should approach, it will be necessary to give to the patient, internally, one-sixth to one-third of a grain, or a subcutaneous injection of one-eighth to one-fourth of a grain of the sulphate of morphine. Though I have but twice been obliged to resort to these means of counteraction, I consider it necessary to have them always on hand.

It will be well to examine the patient some hours after the first application. If the pupil has enlarged considerably, one application daily will soon bring about dilatation, and no further treatment will be necessary, particularly in cases of a non-specific nature. If the enlargement is noticeable, but of little extent, or if there is no change, another application is made with the same care, and the case re-examined the following day. On the second day, those cases which do not present a marked increase of the size of the pupil, are, according to the current rules, subjected to the action of mercury, to depletion, paracentesis of the anterior chamber, or Iridectomy. Those, on the contrary, where the pupil has become larger, are treated with atropine exclusively, and only those where marked constitutional syphilis exists, submitted to a mild mercurial treatment. In this manner a number of patients, in whom it is not easy to determine whether the ocular disease be specific or not, escape the necessity of mercurial medication. I have sometimes found, in mild cases where syphilis existed, the pupil dilated on the second day, and the inflammation diminished: so that, for experiment's sake, I cured the disease locally, before I submitted the patient to an anti-syphilitic course of treatment. The applications of atropine, in substance, may well, in my opinion, supersede all other modes of using this remedy. Their energy and permanence of effect are incontestable. They irritate the eye less than collyria, and the whole of the remedy used comes into action; their use is more convenient than even that of atropine paper; and the dangers, which also exist when collyria are applied *coup sur coup*, do not fall heavy in the balance.

